# An Inducible, Isogenic Cancer Cell Line System for Targeting the State of Mismatch Repair Deficiency

**DOI:** 10.1371/journal.pone.0078726

**Published:** 2013-10-29

**Authors:** Julie M. Bailis, Marcia L. Gordon, Jesse L. Gurgel, Alexis C. Komor, Jacqueline K. Barton, Ilan R. Kirsch

**Affiliations:** 1 Oncology Research, Amgen Inc., South San Francisco, California, United States of America; 2 Oncology Research, Amgen Inc., Seattle, Washington, United States of America; 3 Division of Chemistry and Chemical Engineering, California Institute of Technology, Pasadena, California, United States of America; University of Saarland Medical School, Germany

## Abstract

The DNA mismatch repair system (MMR) maintains genome stability through recognition and repair of single-base mismatches and small insertion-deletion loops. Inactivation of the MMR pathway causes microsatellite instability and the accumulation of genomic mutations that can cause or contribute to cancer. In fact, 10-20% of certain solid and hematologic cancers are MMR-deficient. MMR-deficient cancers do not respond to some standard of care chemotherapeutics because of presumed increased tolerance of DNA damage, highlighting the need for novel therapeutic drugs. Toward this goal, we generated isogenic cancer cell lines for direct comparison of MMR-proficient and MMR-deficient cells. We engineered NCI-H23 lung adenocarcinoma cells to contain a doxycycline-inducible shRNA designed to suppress the expression of the mismatch repair gene MLH1, and compared single cell subclones that were uninduced (MLH1-proficient) versus induced for the MLH1 shRNA (MLH1-deficient). Here we present the characterization of these MMR-inducible cell lines and validate a novel class of rhodium metalloinsertor compounds that differentially inhibit the proliferation of MMR-deficient cancer cells.

## Introduction

Genome instability is a hallmark of cancer cells and can lead to numerical or structural changes to chromosomes (chromosome instability, or CIN), or nucleotide mismatch repair (MMR) deficiency (MIN) [1]. While CIN can result from loss of function of a number of cellular pathways, MIN results specifically from defects in the MMR system and is identifiable by the gain or loss of mono-, di- or tri-nucleotide repeat sequences, referred to as microsatellite instability (MSI) (reviewed in 2). Replication errors such as polymerase slippage generate small insertion-deletion loops (IDLs) or single base mismatches in the DNA. DNA damage can also modify bases to cause mismatches. In human cells that are MMR-proficient, heterodimers that contain the bacterial MutS homolog MSH2 bind a mismatch, and then heterodimers that contain the bacterial MutL homolog MLH1 associate with the protein:DNA complex to mediate the recruitment of repair factors that excise the mismatch and restore the correct DNA sequence. MMR-deficient cells are unable to correct mismatches, resulting in incorporation of errors into the DNA template and a mutator phenotype.

MMR deficiency is strongly linked with cancer. Germ line mutation of MMR genes, particularly MLH1 or MSH2, is the basis of hereditary non-polyposis colorectal cancer (HNPCC), or Lynch syndrome, which confers susceptibility to colorectal cancer but also to other specific cancer types including endometrial and ovarian cancer (reviewed in 3,4). Mutation or hypermethylation of MMR genes in somatic cells is associated with approximately 15% of sporadic colorectal cancers, as well as 10-15% of ovarian, endometrial and gastric cancers (reviewed in 5). MMR deficiency and MSI have also been identified in up to 20% of leukemias in patients that relapse, or that develop the disease as a consequence of prior chemotherapy [6]. 

MMR deficiency and MSI also occur in primary lung cancer associated with smoking or exposure to chromium [7-9]. Cancers with MSI have been reported to be resistant to several standard-of-care chemotherapeutic agents, such as the antimetabolite 5-fluorouracil (5-FU), the platinum compounds cisplatin and carboplatin, the alkylating drug temozolomide, and the topoisomerase inhibitor etoposide [10]. Inactivation of the MMR pathway may allow cells to tolerate certain types of DNA damage without initiating a pathway of programmed cell death [11].

One challenge to identifying therapies for MMR-deficient cancers is that the molecular targets and clinical phenotypes resulting from inactivation of MMR genes are variable. MMR deficiency can lead to frameshift mutations in genes that contain repeat sequences in the DNA, and at least 30 genes have been identified as potential targets of MSI, including the oncogenes BRAF and KRAS, and the DNA damage response genes MRE11, BRCA1 and ATR [12,13]. Efforts to identify novel therapeutic targets that exhibit synthetic lethality with MMR-deficient cancer cells have revealed variability in genetic targets with loss of function of MLH1 or MSH2 [14]. Together, these observations support the idea that cancers with MSI represent a multifaceted, heterogeneous set of diseases. In consideration of the complexity of MSI tumors, we have previously proposed targeting the end phenotype or “state” of mismatch repair deficiency itself [15,16].

In the current effort described here, our goal was to develop tools to enable studies of induced mismatch repair deficiency. We describe a completely isogenic cell line system in which expression of the MMR gene MLH1 can be switched on or off using shRNA. As our model system we used the lung adenocarcinoma NCI-H23, a cell line selected based on relatively high levels of MLH1 that could be reversibly inactivated by shRNA. In this study, we induce MMR deficiency in the NCI-H23 cell line system and demonstrate that this results in MSI and increased resistance to DNA damaging agents. As a potential step toward developing a therapeutic that targets the end “state” of MMR deficiency, we also use this cell line system to further validate a novel class of metal complexes that target DNA mismatches.

## Materials and Methods

### Short hairpin RNA (shRNA)

Sequences predicted to knock down expression of the MLH1 or MSH2 genes were designed using BLOCK-IT RNAi Designer (Life Technologies). Four independent sequences for each gene were chosen as candidate shRNA triggers as indicated below (numbering indicates position on the cDNA).

#### MLH1

362 GCCTGAAGTTGATTCAGATCC

740 GCAGGTATTCAGTACACAATG

928 GGTTACATATCCAATGCAAAC

2237 GCGCTATGTTCTATTCCATCC

#### MSH2

399 GCATCCAAGGAGAATGATTGG

1102 GGATTAAGCAGCCTCTCATGG

1260 GCAGCAAACTTACAAGATTGT

2359 GGGCTATATCAGAATACATTG

The method used for construction of the shRNA cassettes has been described in detail elsewhere [17]. Briefly, shRNA sense-loop-antisense constructs of these sequences were generated by PCR, using TTCATGAGA as the loop sequence. The final PCR product, which contained the sense-loop-antisense shRNA sequences flanked by the *attB1* site and tH1 promoter on the 5’ end, and a termination signal and the *attB2* site on the 3’ end, was then cloned into the Gateway vector pDONR (Life Technologies). Gateway recombination techniques were then used to transfer the shRNA cassettes into Gateway-compatible lentiviral destination vectors pLV736G or pLV739G. 

Lentiviral stocks were prepared as described [17]. 293-METR cells [18] were transfected with a pLV736G or pLV739G vector that contained an individual shRNA cassette, together with plasmids containing the Gag/Pol, Rev, and Env genes. After overnight incubation, the supernatant was collected, filtered, concentrated by ultracentrifugation and stored at -80°C.

### Cell line generation

NCI-H23 cells obtained from the American Type Culture Collection (ATCC) were grown in RPMI media with 10% fetal bovine serum. For lentiviral transduction, NCI-H23 cells were seeded at 2 x 10^4^ cells per ml in 12-well plates and incubated overnight. Media was aspirated from the cells and replaced with Opti-MEM media (Life Technologies) that contained 10 µg/ml Diethylaminoethyl-Dextran (GE Healthcare Life Sciences) and 1-5 x 10^6^ transduction units per milliliter (TU/ml) of lentivirus. Cells were transduced with lentivirus containing MLH1 shRNA or MSH2 shRNA. Plates were incubated overnight, and then media containing lentivirus was aspirated off and replaced with fresh RPMI media. After 1-3 days (d), cells were expanded to 6-well plates and grown in the presence of selection agent (250 µg/ml G418 for MLH1 and 2.5 µg/ml puromycin for MSH2). 500ng/ml doxycycline (Sigma-Aldrich) was added to the cells to induce expression of the shRNA. 

Cells grown under selection and with shRNA induced for at least 2 months were plated to 96-well plates at a density of 0.3 cells per well to select for single cell subclones. Single cell subclones were expanded and maintained under selection conditions. Uninduced cells used for comparison to the induced subclones were obtained by culturing the subclones in the absence of doxycycline for at least one week.

### Antibodies and western blots

Protein lysates were prepared in lysis buffer (50mM HEPES, 1% Triton X-100, 150mM NaCl, 1mM EGTA, 10% glycerol, 1mM dithiothreitol) to which phosphatase and protease inhibitors (20mM -glycerophosphate, 100mM sodium fluoride, 0.5mM phenylmethylsulfonyl fluoride, 0.1mM sodium orthovanadate, 10 µg/ml leupeptin) were added. Lysates were cleared by centrifugation and then analyzed by SDS-PAGE on 4-12% Bis-Tris gels (Life Technologies) followed by immunoblotting. Primary antibodies used were against MLH1 (Becton Dickinson #551091), MSH2 (Becton Dickinson #556349), GAPDH (Abcam #ab9484), phosphorylated histone H2AX (Millipore #05-636) or tubulin (Cell Signaling #2148). Primary antibodies were detected with secondary antibodies conjugated to IRDye700 or IRDye800 (LiCOR) and visualized with the LiCOR Odyssey. Each western blot experiment was carried out at least twice, on independent days. Representative data from a single experiment are shown.

### DNA fragment analysis

Genomic DNA from the NCI-H23 subclones was prepared using a DNeasy kit (Qiagen). Multiplex PCR reactions were carried out according to the MSI Analysis System, Version 2.1 (Promega). PCR products were analyzed on an ABI 3130 sequencer, and then the data were analyzed using GeneMapper software (Life Technologies). Subclones that exhibited MSI for at least one marker in the first experiment were retested in an independent experiment to confirm the results.

### Cell viability assays

Cells grown in the absence or presence of doxycycline were plated at 2000-5000 cells per well in 96-well plates and allowed to incubate overnight. Cells were then treated with compounds in a dose response for 4d. Cell viability was assessed using a Cell Titer-Glo assay (Promega) for ATP metabolism. Cell viability assays were performed in duplicate in at least 3 independent experiments. Representative data from one such experiment are shown. 

Phase contrast imaging of cells during the proliferation experiments was carried out using an IncuCyte with a 20X objective (Essen BioScience). 

Colony forming assays were also used to test cell viability following compound treatment. Cells grown in the absence or presence of doxycycline were plated at 500-2000 cells per well of a 6-well plate and allowed to incubate overnight. Cells were treated with compounds in a dose response for 24 hours (h), and then media was aspirated and replaced with fresh media that did not contain compound. Colonies were visualized with crystal violet stain at 10-12d.

Senescence associated beta-galactosidase (SA--gal) production was assessed using a histochemical stain for beta-galactosidase activity at pH6.0 (Sigma-Aldrich). Cells were untreated or treated with compounds as indicated for 3d, and then cells were fixed, stained for SA--gal and imaged using a Zeiss Axio inverted microscope equipped with a color camera.

### Statistical analysis

For the cell viability assays, paired t tests were used to compare half maximal inhibitory (IC_50_) or median lethal dose (LD_50_) values from multiple experiments and determine p values. P values equal to or less than 0.05 were considered statistically significant. Statistical analysis was done with Graph Pad Prism software.

### Microarray analysis

Total RNA was prepared from duplicate samples of cells in log phase of growth, using an RNeasy mini kit (Qiagen). Sample integrity was assessed on a BioAnalyzer. Cy3- and Cy5-labeled RNA was prepared from 200ng total RNA per sample, and then hybridized to Human Whole Genome Arrays (Agilent Technologies) according to the Agilent Two-Color Microarray-Based Gene Expression Analysis Protocol. Data were analyzed in Rosetta Resolver. 

### Chemical compounds

Synthesis of [Rh (HDPA)_2_chrysi]^3+^ and [Rh(DIP)_2_chrysi]^3+^ have been described [19,20]. The synthesis of [Rh(DPE)(phen)(chrysi)]^3+^ was recently reported [21]. Cisplatin, doxorubicin, etoposide, 6-thioguanine and temozolomide were obtained from Sigma-Aldrich and dissolved in DMSO or water. The CDK4/6 inhibitor PD-0332991 [22], used as a positive control for senescence assays, was synthesized according to published methods.

## Results

### Inhibition of MLH1 by shRNA reduces MLH1 protein levels

The NCI-H23 lung adenocarcinoma cell line is proficient for mismatch repair and contains relatively high levels of the MMR proteins MLH1 and MSH2 [23]. With the goal of generating a matched system that induces MMR deficiency and allows direct comparison to MMR-proficient cells, we tested whether expression of these MMR proteins could be downregulated by shRNA. NCI-H23 cells were transduced with lentivirus containing inducible shRNA to MLH1 or MSH2, and cells that stably integrated the construct into the genome were selected and expanded. Under normal growth conditions, the integrated shRNA was not active and the MMR proteins continued to be expressed. Expression of the shRNA was regulated by treating the cells with doxycycline. When the shRNA was induced, protein lysates from cells that contained any of the four shRNA constructs against MLH1 demonstrated near complete (>90%) inhibition of the MLH1 protein (Figure S1). In contrast, the MSH2 protein was only partially decreased after induction of any of the four MSH2 shRNA constructs in NCI-H23 cells (Figure S1) and these cells were not further characterized. 

**Figure 1:**
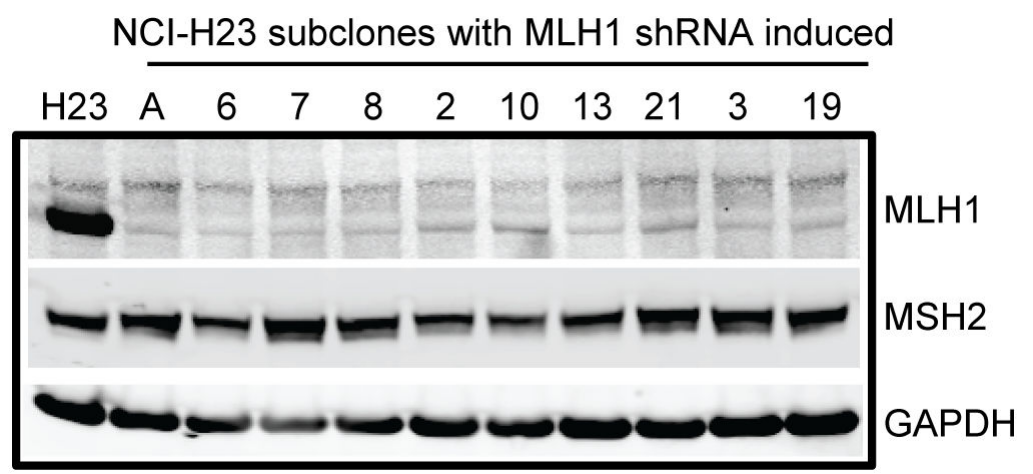
Induction of MLH1 shRNA decreases MLH1 protein levels. MLH1 protein levels in NCI-H23 subclones induced for the MLH1 928 shRNA were compared to MLH1 protein levels in the NCI-H23 parental cells (H23). Protein lysates were analyzed by SDS-PAGE and immunoblotting for the MLH1 and MSH2 protein levels. GAPDH was used as a control for protein loading.

 Because of the potential for variability of shRNA expression within a cell population, we isolated and characterized single cell subclones from NCI-H23 cells transduced with one of the shRNA constructs, MLH1 928. Cells were grown in the presence of doxycycline to induce the MLH1 shRNA for at least one month, and then plated for single cells which were expanded into colonies that were maintained in inducing conditions. MMR protein levels were then re-assessed by immunoblotting. The NCI-H23 subclones that expressed MLH1 shRNA consistently reduced the levels of MLH1 protein >90%, without affecting the levels of MSH2 protein (Figure 1). 

### Downregulation of MLH1 induces microsatellite instability

 We selected at least 20 subclones from NCI-H23 cells that expressed MLH1 shRNA to test for MSI. Genomic DNA was prepared from parental NCI-H23 cells and from each subclone, and then multiplex PCR was carried out to amplify a standard panel of microsatellite markers (BAT-26, BAT-25, MONO-27, NR-21 and NR-24). Fragment sizes of the PCR products were then analyzed on a DNA sequencer. All of the NCI-H23 subclones showed MSI at the mononucleotide repeat BAT-26 [24], indicated by a shift of 1-3 nucleotides at the locus (Figure S2). MLH1 deficiency is therefore sufficient to induce MSI. A number of the subclones also displayed possible MSI at another marker, with independent subclones demonstrating alteration of different markers (Figure S2). Subclones 4-10 and 4-13, which were obtained by transduction of NCI-H23 cells with the MLH1 928 shRNA construct, were chosen for additional phenotypic analysis.

### Downregulation of MMR genes by shRNA is reversible

**Figure 2:**
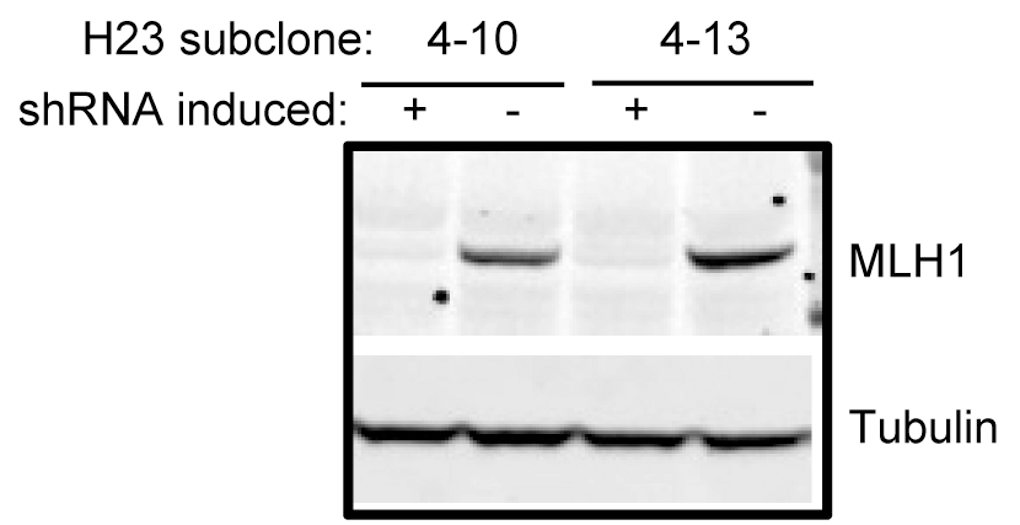
MLH1 shRNA expression is inducible and reversible. NCI-H23 subclones 4-10 and 4-13 that were induced for MLH1 shRNA were split into two cultures, one maintained in inducing conditions (+ doxycycline) and the other grown in the absence of doxycycline (-) to allow re-expression of MLH1. Protein lysates were prepared and analyzed by SDS-PAGE followed by immunoblotting for expression of the MLH1 protein. Tubulin was used as a control for equal protein loading across samples.

The NCI-H23 single cell subclones were maintained under growth conditions that continually induced shRNA expression. To test whether the shRNA-mediated downregulation of MLH1 was reversible, cells from each subclone were split into two separate cultures, with one culture maintained in the presence of doxycycline to maintain MLH1 deficiency, and the other culture grown in the absence of doxycycline to allow MLH1 expression. After one week, protein lysates were prepared from the cells, and the MLH1 protein level was assessed by immunoblotting. In the absence of doxycycline, cells from the 4-10 and 4-13 subclones were able to re-express wild-type levels of MLH1 protein (Figure 2). Not all of the subclones tested were able to re-express MLH1 protein after growth in the absence of doxycycline (data not shown), suggesting that the shRNA in those subclones might be constitutively expressed.

### MLH1-deficient NCI-H23 subclones do not display global changes in gene expression

Microarray analysis was carried out on the NCI-H23 single cell subclones to test whether downregulation of MLH1 by shRNA affects the expression of other genes. Cells from subclones 4-10 and 4-13 were either maintained in the presence of doxycycline to induce MLH1 shRNA, or grown in the absence of doxycycline for at least a week to allow re-expression of MLH1. The parental cell line NCI-H23, either untreated or treated with doxycycline, was included as a control. Total RNA was isolated from duplicate samples of cells, fluorescently labeled, and hybridized to Agilent whole genome arrays. Gene expression patterns in the uninduced subclones were similar to those of the parental NCI-H23 cells, either untreated or treated with doxycycline (data not shown). Levels of the MLH1 gene transcript were decreased approximately three-fold in the 4-10 and 4-13 subclones where MLH1 shRNA had been induced (Figure S3). A more widespread gene expression signature associated with MLH1 shRNA induction did not emerge from the data (Figure S3). 

### MLH1-deficient NCI-H23 subclones exhibit increased resistance to chemotherapeutic drugs

**Figure 3:**
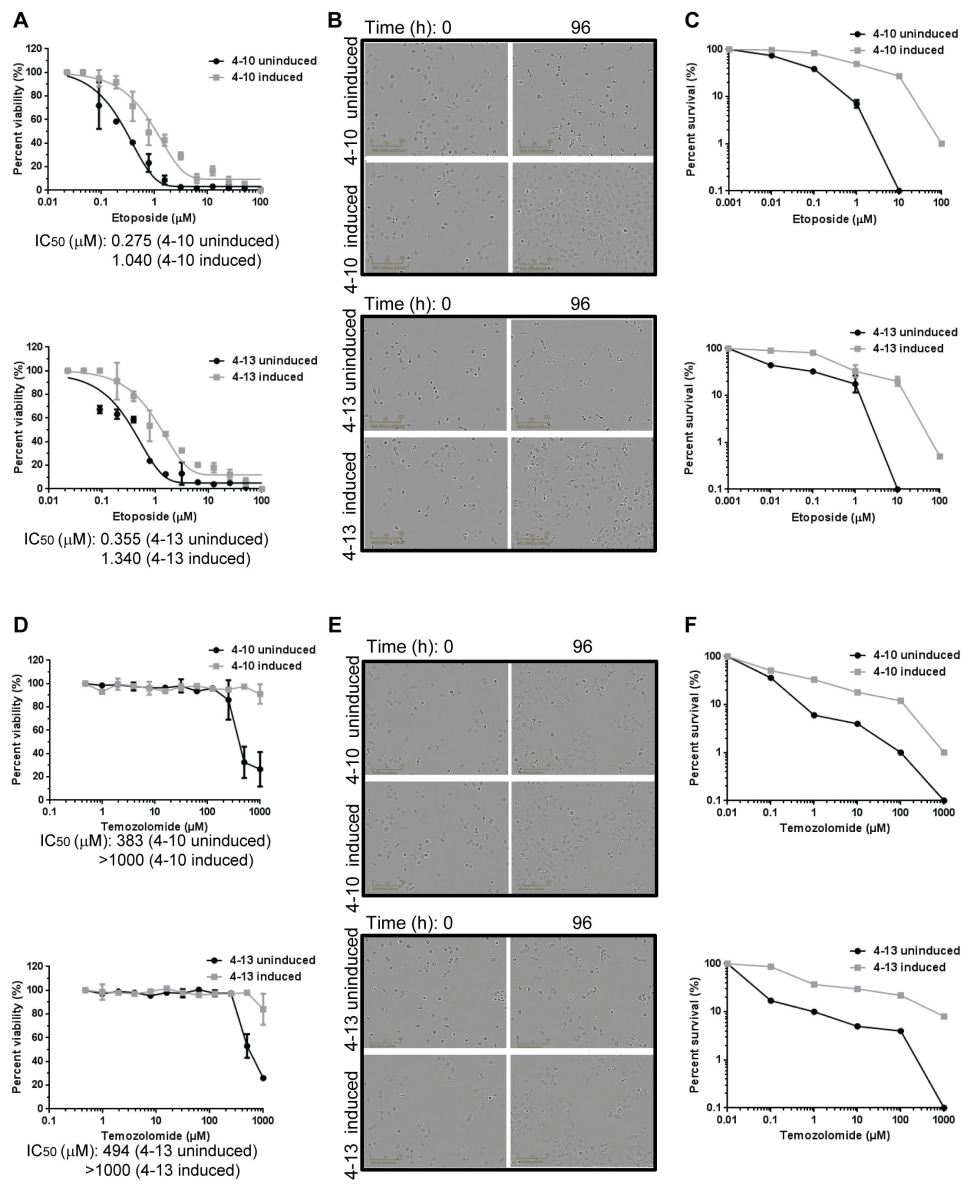
MLH1-deficient NCI-H23 subclones exhibit increased resistance to chemotherapeutic drugs. NCI-H23 subclones that were uninduced or induced for MLH1 shRNA were treated with etoposide (A-C) or temozolomide (D-F). (A, D) Cells were treated with compound at the concentrations indicated, and cell viability was assessed at 4d using a Cell Titer-Glo assay. The graphs indicate the relative survival for duplicate samples from a single experiment. T tests to compare IC_50_ values across multiple experiments determined p values of p=0.04 for cells treated with etoposide, and p=0.01 for cells treated with temozolomide. (B, E) Phase contrast images of cells at 0h and 96h time points during the cell viability experiment are shown. Cells were treated with 390 µM etoposide (B) or 500 µM temozolomide (E). (C, F) Cells were treated with compound for 24h at the concentrations indicated, and colony forming ability after compound washout was assessed. The graphs display percent survival at 10d for duplicate samples of cells treated with etoposide (C) or temozolomide (F). Comparison of the LD_50_ values across multiple experiments by t-test determined p values of p=0.03 for cells treated with etoposide, and p=0.04 for cells treated with temozolomide.

We next tested whether induced MMR deficiency would cause resistance to DNA damaging agents. We directly compared the MMR-deficient NCI-H23 subclones to MMR-proficient cells from the same subclone that were obtained by allowing the cells to re-express MLH1. The MMR-deficient and MMR-proficient subclones were treated with the topoisomerase inhibitor etoposide or the alkylating agent temozolomide and cell viability was assessed after 4d (Figure 3A, D). The MMR-proficient subclones, in which the shRNA was uninduced, did not show a significant difference in sensitivity to the compounds compared to parental NCI-H23 cells that were either untreated or treated with doxycycline (p=0.34) (Figure S4). For each matched pair of cells (for example, subclone 4-10 uninduced compared to 4-10 induced), the MLH1-deficient cells were consistently at least two-fold more resistant to these compounds than the isogenic MLH1-proficient cells, a finding that is statistically significant (p=0.04 for cells treated with etoposide, and p=0.01 for cells treated with temozolomide) (Figure 3A, D). The MLH1-deficient subclones were also more resistant to the crosslinking agent cisplatin (p=0.02), the purine analog 6-thioguanine (p=0.05), and another topoisomerase inhibitor, doxorubicin (p=0.04) (Figure S5). The approximately two-fold difference in viability of MMR-deficient cells versus MMR-proficient cells in vitro in response to chemotherapeutic agents has been reported previously and shown to translate to drug resistance in vivo (reviewed in 4,10). To confirm that the differential effect on viability is due to the presence or absence of MLH1, we used different shRNA sequences, 362 and 2239, to inhibit MLH1 expression and then characterized cell sensitivity to chemotherapeutic drugs. We observed that the MLH1-deficiency resulting from induction of these independent shRNA triggers led to a similar increase in resistance to chemotherapeutic drugs relative to the NCI-H23 cells that expressed MLH1 (p=0.01) (Figure S6). 

 To explore the differential sensitivity to the compounds in more detail, we imaged cells over the 4d treatment period of the cell viability assay and examined cell count and morphology. At the 0h time point, the cells are in growth phase and appear healthy by phase contrast imaging. However by 96h, the differences between the uninduced and induced subclones were apparent: most of the MLH1-proficient cells had undergone cell death after treatment with the DNA damaging agents, whereas the MLH1-deficient cells continued to proliferate (Figure 3B, E). Treatment with the DNA damaging agents did not affect MLH1 expression in the uninduced subclones but did result in increased expression of phosphorylated histone H2AX, a marker for DNA fragmentation [25], suggesting that the MLH1-expressing cells had undergone apoptosis (Figure S7). 

 We further investigated cell survival following compound treatment using clonogenic assays. Cells were treated with etoposide or temozolomide for 24h, and recovery from compound treatment was assessed by colony formation after 10d. The MLH1-deficient NCI-H23 subclones were more resistant to compound treatment than the isogenic cells proficient for MLH1 (Figure 3C, F), showing a significant difference in the LD_50_ values (p=0.03 for etoposide, and p=0.04 for temozolomide). Together, these results demonstrate a differential response to DNA damage that is based solely on the presence or absence of MLH1.

### MLH1-deficient NCI-H23 subclones display increased sensitivity to rhodium metalloinsertor compounds

We previously described metal complexes that noncovalently bind DNA mismatches with moderate affinity and high specificity, due to thermodynamic destabilization of the mismatched base pairs (reviewed in 26). We initially demonstrated that this class of compounds preferentially inhibits the proliferation of MMR-deficient cells, using a matched HCT-116 colorectal cancer cell line system and a genetic knockout system [16,19,21]. This earlier work was the foundation and motivation for the creation of the isogenic cell lines described in the current study and led us to test whether rhodium metalloinsertor compounds would also preferentially inhibit the viability of these cells when MMR deficiency was induced.

**Figure 4:**
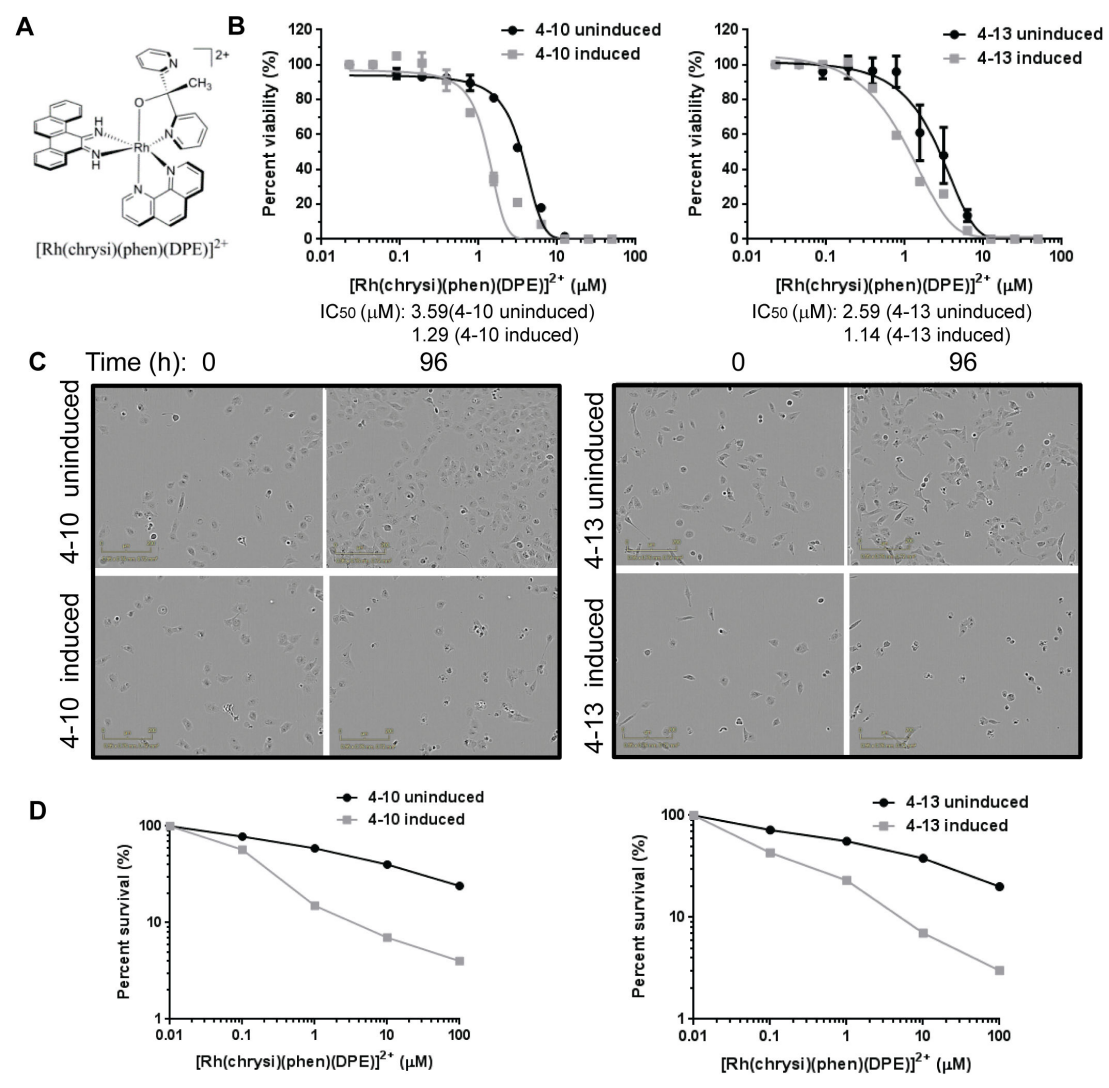
MLH1-deficient NCI-H23 cells display increased cell sensitivity to rhodium metalloinsertor compounds. NCI-H23 subclones that were uninduced or induced for MLH1 shRNA were treated with the rhodium metalloinsertor compound [Rh(chrysi)(phen)(DPE)]^3+^. (A) Cells were treated at concentrations indicated, and cell viability was assessed after 4d using a Cell Titer-Glo assay. Percent viability of duplicate samples from a single experiment is shown. The p value was determined as p=0.01 by a t test. (B) Phase contrast images of cells treated with 5 µM [Rh(chrysi)(phen)(DPE)]^3+^ at 0h and 96h during the cell viability assay are shown. (C) Cells were treated with [Rh(chrysi)(phen)(DPE)]^3+^ for 24h, and then assayed for colony formation after 10d. The graphs show percent survival of duplicate samples of compound treated cells. The p value was determined as p=0.01 by a t test.

 In our inducible cell line system, the rhodium metalloinsertor compound [Rh(chrysi)(phen)(DPE)]^3+^ preferentially inhibited the viability of induced MLH1-deficient NCI-H23 subclones, with at least a three-fold change in the cellular IC_50_ in a 4d Cell Titer-Glo assay relative to the MLH1-proficient subclones (p=0.01) (Figure 4A, B). Another rhodium metalloinsertor compound, [Rh(HDPA)_2_chrysi]^3+^ also preferentially inhibited proliferation of MLH1-deficient cells (Figure S8), with a two- to three-fold change in cellular IC_50_ values (p=0.02). To confirm that the difference resulted from MLH1 deficiency rather than an off-target effect of the shRNA, we used additional, independent MLH1 shRNA constructs to downregulate MLH1 in NCI-H23 cells and verified that the MLH1-deficient cells were preferentially sensitive to rhodium metalloinsertor compounds (p=0.01) (Figure S9). In contrast, a related compound that exhibits only weak binding to mismatched DNA, [Rh(DIP)_2_chrysi]^3+^ [21] did not display a differential effect on cell viability of the uninduced and induced NCI-H23 clones (p=0.90) (Figure S9). Together, our data support the hypothesis that downregulation of MLH1 increases cell sensitivity to the rhodium metalloinsertor compounds.

We examined the cellular morphology of the NCI-H23 subclones treated with [Rh(chrysi)(phen)(DPE)]^3+^ during the time course of the proliferation assays, using phase contrast imaging. Cells from the MLH1-proficient and MLH1-deficient subclones appeared healthy and in growth phase at the 0h time point. By 96h, the uninduced MLH1-proficient cells had continued to proliferate but fewer cells appeared to be undergoing mitosis, suggesting a cell cycle delay or arrest (Figure 4C). The cells did not appear to be senescent, as evidenced by lack of production of SA--gal [26] (data not shown). In contrast, most of the MLH1-deficient cells either failed to proliferate, or had undergone cell death by 96h (Figure 4C). We verified that this differential effect was due to the absence or presence of MMR in the cells by assessing MLH1 protein levels in cells treated with rhodium metalloinsertor compounds (Figure S10). The previously demonstrated effect of shRNA induction on MLH1 protein expression was not altered following treatment with the rhodium compounds (Figure S10).

We also confirmed the increased sensitivity of the MLH1-deficient NCI-H23 subclones to the rhodium metalloinsertor compounds using clonogenic assays. After 24h treatment with [Rh(chrysi)(phen)(DPE)]^3+^, the MLH1-deficient NCI-H23 subclones formed fewer colonies than the isogenic MLH1-proficient subclones, exhibiting a significant difference in LD_50_ values (p=0.01) (Figure 4D). The differential sensitivity of the MLH1-deficient subclones to the rhodium metalloinsertor compounds is consistent with the hypothesis that these compounds exert their effects through binding to DNA mismatches that are present in MMR-deficient cells.

## Discussion

 The MMR pathway maintains genome stability by promoting the recognition and repair of single base mismatches and small insertion-deletion loops in the DNA that result from replication errors or DNA damage. Loss of function of the MMR pathway increases the prevalence of cellular mutations and can cause or contribute to cancer. The mechanism of carcinogenesis resulting from inherited MMR deficiency has been well studied, particularly in colorectal cancer [4,27], and has been modeled in cell line systems such as HCT-116 O cells, which are MLH1-deficient but contain an extra copy of Chromosome 2, and HCT-116 N cells, in which the MLH1 deficiency is complemented by an extra copy of Chromosome 3 that contains wild type MLH1 [28]. In other cancer types, such as in lung cancer, MMR deficiency can be promoted by carcinogen exposure [7-9]. Chemotherapy treatment also may promote MMR deficiency leading to secondary leukemia [6]. The cell line system we describe provides the first example of an isogenic model for induced MMR deficiency that can be reversibly switched on or off at the level of control of protein expression. It provides a model to study questions about induced MMR deficiency and to allow identification of molecules that might offer therapeutic benefit in MMR-deficient cancers. 

The NCI-H23 matched cell line system described here permits detailed characterization of the timing and sequence of events that result when MMR deficiency is induced. We observed similar effects with three different shRNA sequences targeted to MLH1, and in two different single cell subclones isolated from cells transduced with one of the MLH1 shRNA constructs. ShRNA-mediated inhibition of MLH1 expression is an early event in induced MMR deficiency, with MLH1 protein levels nearly undetectable by 3d. In contrast, the genomic alterations resulting from MLH1 loss of function in cells (for example, MSI) were not observed until several additional weeks in culture. Consistent with reports that MMR deficiency may lead to mutation of different target genes in different individuals or cancer types [12,13,29], we found that independent cell subclones displayed MSI at different molecular markers. Re-expression of MLH1 by growth of the subclones in the absence of doxycycline restores MMR function and provides the possibility that re-activation of the MMR pathway in cancer cells can also be investigated using this cell line system. Further studies with the inducible NCI-H23 subclones may facilitate understanding of drug response and resistance in MMR-deficient cancer cells. 

Our system is complementary to matched cell line systems commonly used to study MMR, such as the MMR-deficient cancer cell lines HCT-116 or Hec59, which are complemented by an extra copy of the chromosome containing wild type MLH1 or MSH2, respectively [28,30]. In these models, the MMR-proficient cells and MMR-deficient cells are generated as different clones, and are chromosomally distinct from each other and the parental cell line. These differences may result in changes in chromosome stability or gene expression that are not solely due to MMR deficiency [31,32]. Matched normal cell systems, such as MMR-deficient embryonic kidney cells 293T compared to 293T cells engineered to overexpress MLH1 [33], or wild-type mouse embryonic fibroblasts (MEFs) compared to MSH2-deficient MEFs [34] may be not be ideal models to study MMR function in cancer. MEFs may be less sensitive to defects in DNA repair than cancer cells, and have been reported to exhibit a much lower mutation rate than MMR-deficient cancer cells [31,35].

There is currently no targeted therapy for patients with MMR-deficient cancer. The standard of care for patients with colorectal cancer remains adjuvant combined chemotherapy that includes the nucleoside inhibitor 5-FU, even though multiple clinical studies have now shown that MMR-deficient tumors might not benefit from 5-FU treatment (reviewed in 4,27). In addition to defects in DNA damage recognition and repair [11], MMR-deficient colorectal tumors display distinct features such as localization to the proximal colon, decreased metastasis, increased number of tumor infiltrating lymphocytes, and near-diploid DNA content of the cells [36]. These characteristics may contribute to the better overall prognosis reported for patients with MMR-deficient colorectal cancer when compared on a stage by stage basis to patients with MMR-proficient cancers [37]. However, there is still a need for therapeutics that will be efficacious in MMR-deficient tumors. 

Synthetic lethality screens have identified several potential therapeutic targets for MMR-deficient cancer cells. Methotrexate, an inhibitor of dihydrofolate reductase, was reported to show synthetic lethality in MSH2-deficient cancer cells [38] and is currently in clinical trials for MSH2-deficient colorectal cancer (www.clinicaltrials.gov; Identifier: NCT00952016). Inhibition of DNA polymerases, POLB or POLG, caused synthetic sickness or synthetic lethality in cancer cells deficient for MSH2 or MLH1, respectively [38]. MMR-deficient colorectal cancer cells were also reported to be preferentially sensitive to inhibitors of cytosine-based nucleoside analogs such as cytarabine [39], the PI3 kinase/AKT pathway [40], and the PTEN-induced putative kinase PINK1 [41]. A disadvantage of the synthetically lethal interactions identified is that in several cases, these appear to be specific for a single gene mutation and may not apply to targets in other pathways. It remains to be determined whether the proposed mechanistic basis for these synthetically lethal interactions will translate to efficacy in patients with genotypic and phenotypic tumor heterogeneity.

We previously considered targeting the end “state” of genomic instability [15]. We have demonstrated that rhodium metalloinsertor compounds can bind DNA mismatches and preferentially inhibit the proliferation of MMR-deficient cells, including the MLH1-deficient HCT-116 colorectal cancer cell line [16,19,21]. Here we confirm and extend our previous observations by demonstrating, in a completely isogenic system, preferential sensitivity of cells induced for MMR deficiency to rhodium metalloinsertor compounds. The differential activity of the compounds in MMR-deficient cells correlates with their binding affinity to DNA mismatches in vitro [19]. Recently, the differential cellular activity of these compounds has also been shown to correlate with their accumulation in the cell nucleus [21]. Together, these observations support the model that rhodium metalloinsertor compounds act directly on DNA mismatches in genomic DNA. The metal complexes enter cells by passive diffusion and bind non-covalently to mismatched DNA due to the thermodynamic destabilization of mispaired nucleosides (reviewed in 42). This mechanism of mismatched base pair recognition is distinct from the checkpoint surveillance process normally used by cells which involves proteins associated with the replication fork during S phase [43]. MMR-deficient cells show a 100- to 1000-fold increase in spontaneous mutation rate (1 X 10^-6^ to 1 X 10^-8^) compared to that of MMR-proficient cells which is estimated at 1 X 10^-9^ to 1 X 10^-10^ per replicated base pair (reviewed in 44,45). It is provocative that the rhodium metalloinsertor compounds have the ability to recognize this number of DNA mismatches, and differentially inhibit the proliferation of MMR-deficient cells in vitro, in the context of approximately 6 billion correctly paired bases. We speculate that this differential effect may translate to even greater therapeutic benefit in vivo.

It is striking that the MLH1-deficient cells, which show increased resistance to DNA damaging agents, are preferentially sensitive to rhodium metalloinsertor compounds. We have observed differential effects for this class of compounds consistently across multiple experiments and in different assays for cell proliferation. The differential effect on cell proliferation observed with the rhodium metalloinsertor compounds cannot be explained simply by the growth rate of the MLH1-deficient NCI-H23 subclones, which is similar for the MLH1-proficient and the MLH1-deficient cells. We do not yet understand the molecular mechanism of the preferential sensitivity of MMR-deficient cells to rhodium metalloinsertor compounds. The compounds might interfere with cellular DNA replication or transcription. The replication fork may not be able to synthesize through mismatched DNA bound with a metal complex However, the metal complexes bind noncovalently to mismatched DNA, suggesting that the compounds may dissociate during the DNA unwinding that occurs during replication. Alternatively, the compounds might associate with proteins adjacent to a DNA mismatch. Current studies are focused on addressing these hypotheses, and also on improving the potency and drug-like properties of the current series of rhodium metalloinsertor compounds for applications in cancer therapy. 

## Supporting Information

Figure S1 **Multiple, independent MLH1 shRNA constructs can downregulate MLH1 protein.** NCI-H23 cells were transduced with 4 independent shRNA constructs against MLH1 or MSH2 and then maintained with the shRNA uninduced (-), or treated with 1 µg/ml doxycycline to induce shRNA expression (+). Protein lysates were analyzed by SDS-PAGE and immunoblotting for (A) MLH1 or (B) MSH2 protein. Tubulin levels were used as a control for equal protein loading across samples.
(TIF)

Figure S2 **Single cell subclones with MLH1 shRNA induced exhibit microsatellite instability.** Genomic DNA was prepared from MLH1-deficient NCI-H23 subclones and NCI-H23 parental cells (H23) and used in multiplex PCR for 5 standard markers of MSI. MSI was determined by fragment analysis. Clones 4-10 and 4-13 displayed microsatellite instability at the BAT-26 marker. Clone 4-10 also displayed possible microsatellite instability at the NR-21 marker, while clone 4-13 showed possible instability at the MONO-27 and NR-21 markers. Each clone was analyzed at least twice in independent experiments; representative data from a single experiment are shown.
(TIF)

Figure S3 **Microarray analysis of NCI-H23 subclones.** Total RNA from NCI-H23 subclones grown under uninducing or inducing conditions was labeled and hybridized to whole genome arrays. The gene expression data was exported to Rosetta Resolver and trends compared between the uninduced and induced samples and between the different subclones. The graph shows a comparison of the changes in gene expression for the 4-10 subclone versus (vs.) the 4-13 subclone. Genes with no change in expression level between the subclones are marked in blue. The MLH1 gene, which showed a three-fold decrease in expression in the MLH1-deficient subclones, is indicated in red text.
(TIF)

Figure S4 **Uninduced NCI-H23 subclones show similar sensitivity to etoposide as the parental cells.** NCI-H23 subclones that were uninduced (- Dox) were compared to parental NCI-H23 cells grown in the presence (+ Dox) or absence (- Dox) of doxycycline. Cells were treated at concentrations indicated, and cell viability was assessed after 4d using a Cell Titer-Glo assay. Percent viability of single samples from a representative experiment is shown. Comparison of the IC_50_ values by t test determined that p=0.34.
(TIF)

Figure S5 **MLH1-deficient NCI-H23 subclones display increased resistance to DNA-damaging drugs.** NCI-H23 subclones that were uninduced or induced for MLH1 shRNA were treated with (A) cisplatin, (B) 6-thioguanine or (C) doxorubicin as indicated, and then cell viability was assessed after 4d using a Cell Titer-Glo assay. The graphs indicate the relative survival for duplicate samples from a single experiment. T tests determined the p values as p=0.02, p=0.05 and p=0.04, respectively, for cells treated with cisplatin, 6-thioguanine or doxorubicin.
(TIF)

Figure S6 **Independent MLH1 shRNA constructs confer differential sensitivity to etoposide.** NCI-H23 cells transduced with (A) MLH1 shRNA 362 or (B) MLH1 shRNA 2239 were divided into two cultures, and grown in conditions that were uninduced (MLH1-proficient) or induced for MLH1 shRNA (MLH1-deficient). The cells were treated with etoposide and cell viability was assessed after 4d using a Cell Titer-Glo assay. Percent viability of duplicate samples from a representative experiment is shown. The p value was determined as p=0.01 by t test.
(TIF)

Figure S7 **Etoposide treatment induces apoptosis in MMR-proficient NCI-H23 subclones.** MLH1-proficient and MLH1-deficient NCI-H23 subclones were treated with 10 µM etoposide for 24h, and then levels of phosphorylated histone H2AX (Phospho-H2AX), a marker for apoptosis, were assessed. MLH1 protein is shown to confirm the cells are MMR-proficient or MMR-deficient. Tubulin levels are shown as a control for protein loading.
(TIF)

Figure S8 **Sensitivity of NCI-H23 subclones to additional rhodium metalloinsertor compounds.** (A) Chemical structure of [Rh(HDPA)_2_chrysi]^3+^. (B) NCI-H23 subclones that were uninduced or induced for MLH1 shRNA were treated with [Rh(HDPA)_2_chrysi]^3+^ as indicated, and cell viability was assessed after 4d using a Cell Titer-Glo assay. A t test determined the p value to be p=0.02. (C) Chemical structure of [Rh(DIP)_2_chrysi]^3+^. (D) NCI-H23 subclones that were uninduced or induced for MLH1 shRNA were treated with [Rh(DIP)_2_chrysi]^3+^ as indicated, and cell viability was assessed after 4d using a Cell Titer-Glo assay. Percent viability from duplicate samples of a single experiment is shown. A t test determined the p value to be p=0.90.
(TIF)

Figure S9 **Independent MLH1 shRNA constructs cause preferential sensitivity to rhodium metalloinsertor compounds.** NCI-H23 cells transduced with MLH1 shRNA 362 or MLH1 shRNA 2239 were divided into two cultures, and grown in conditions that were uninduced (MLH1-proficient) or induced for MLH1 shRNA (MLH1-deficient). Cells were treated with (A) [Rh(DPE)(phen)chrysi]^3+^ or (B) [Rh(HDPA)_2_chrysi]^3+^ and cell viability was assessed after 4d using a Cell Titer-Glo assay. Percent viability from duplicate samples of a single experiment is shown. The p value was determined as p=0.01 by t test.
(TIF)

Figure S10 **Treatment of NCI-H23 subclones with rhodium metalloinsertor compounds does not alter MSI status.** MLH1-proficient and MLH1-deficient NCI-H23 subclones were treated with 5uM [Rh(DPE)(phen)chrysi]^3+^ for 24h, and then protein lysates were prepared and analyzed for MLH1 protein levels as a marker for MSI. Levels of tubulin were also assessed as a control for protein loading.
(TIF)
