# Supplementary figures and images for: An Inducible, Isogenic Cancer Cell Line System for Targeting the State of Mismatch Repair Deficiency
Source: PLoS One. 2013 Oct 29;8(10):e78726. doi: 10.1371/journal.pone.0078726 (PMC3812133; doi:10.1371/journal.pone.0078726)

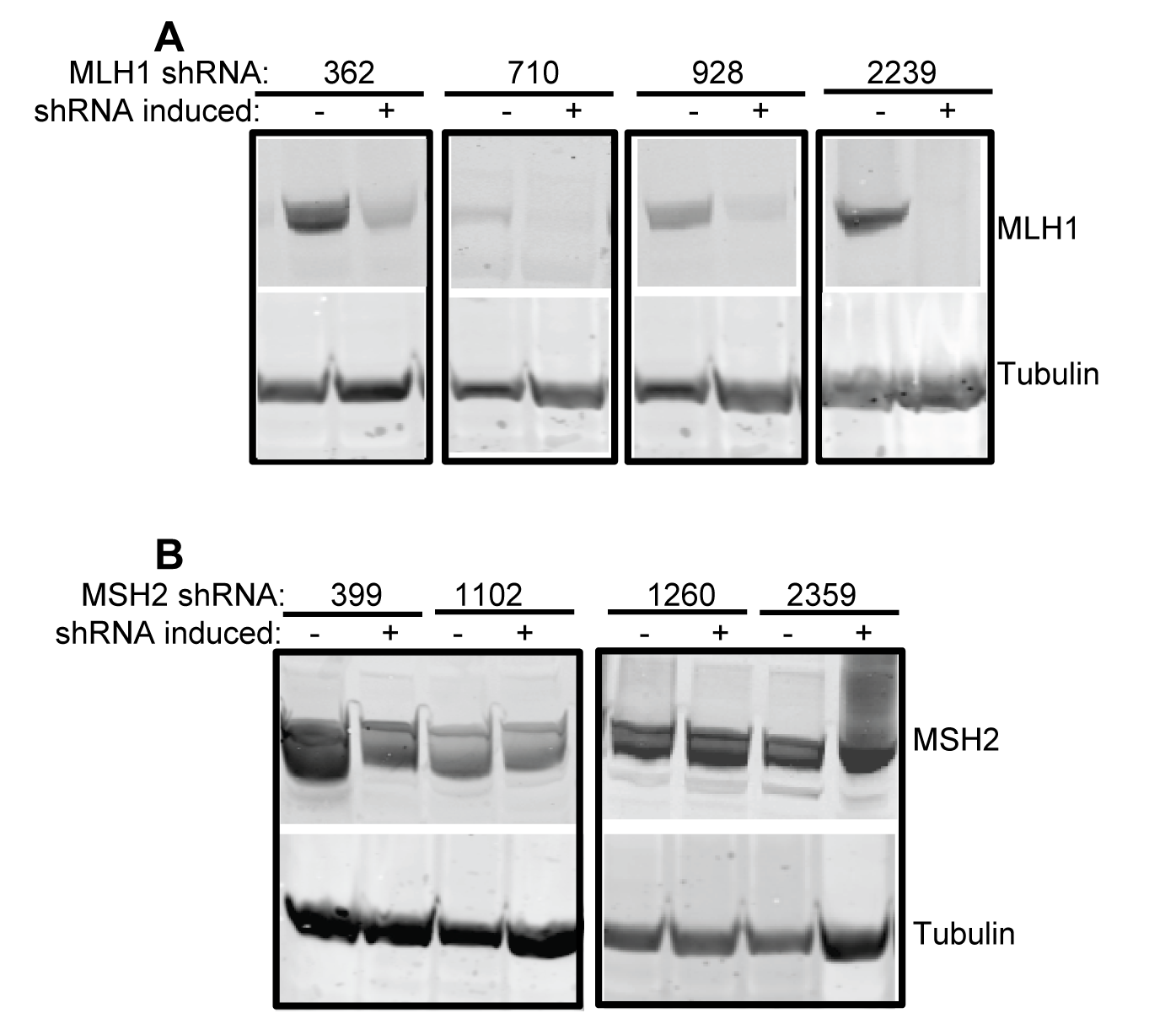

Supplement: Figure S1 — Multiple, independent MLH1 shRNA constructs can downregulate MLH1 protein. NCI-H23 cells were transduced with 4 independent shRNA constructs against MLH1 or MSH2 and then maintained with the shRNA uninduced (-), or treated with 1 µg/ml doxycycline to induce shRNA expression (+). Protein lysates were analyzed by SDS-PAGE and immunoblotting for (A) MLH1 or (B) MSH2 protein. Tubulin levels were used as a control for equal protein loading across samples. (TIF) [file pone.0078726.s001.tif]

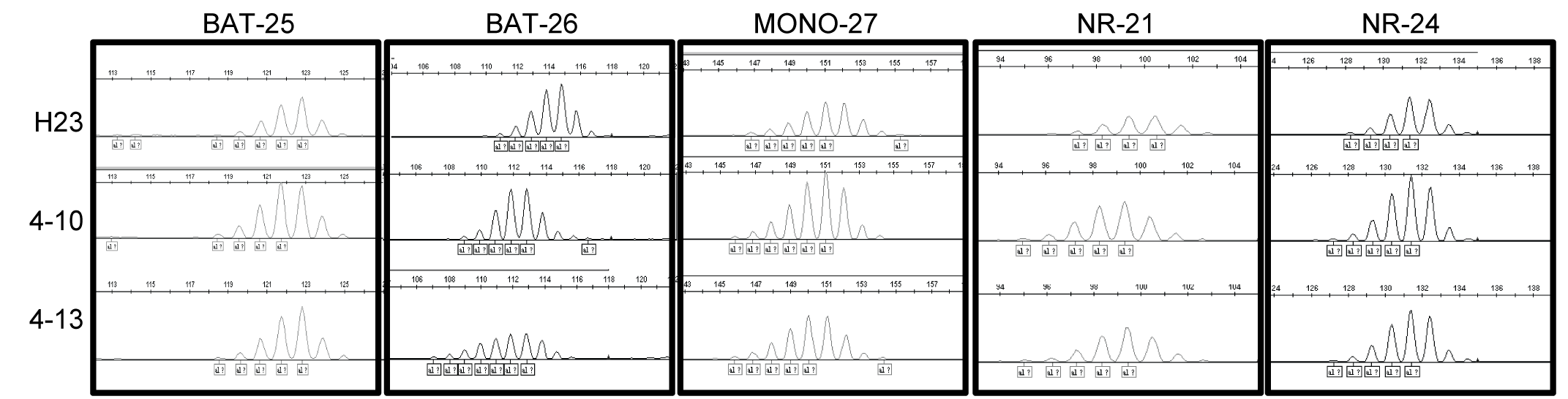

Supplement: Figure S2 — Single cell subclones with MLH1 shRNA induced exhibit microsatellite instability. Genomic DNA was prepared from MLH1-deficient NCI-H23 subclones and NCI-H23 parental cells (H23) and used in multiplex PCR for 5 standard markers of MSI. MSI was determined by fragment analysis. Clones 4-10 and 4-13 displayed microsatellite instability at the BAT-26 marker. Clone 4-10 also displayed possible microsatellite instability at the NR-21 marker, while clone 4-13 showed possible instability at the MONO-27 and NR-21 markers. Each clone was analyzed at least twice in independent experiments; representative data from a single experiment are shown. (TIF) [file pone.0078726.s002.tif]

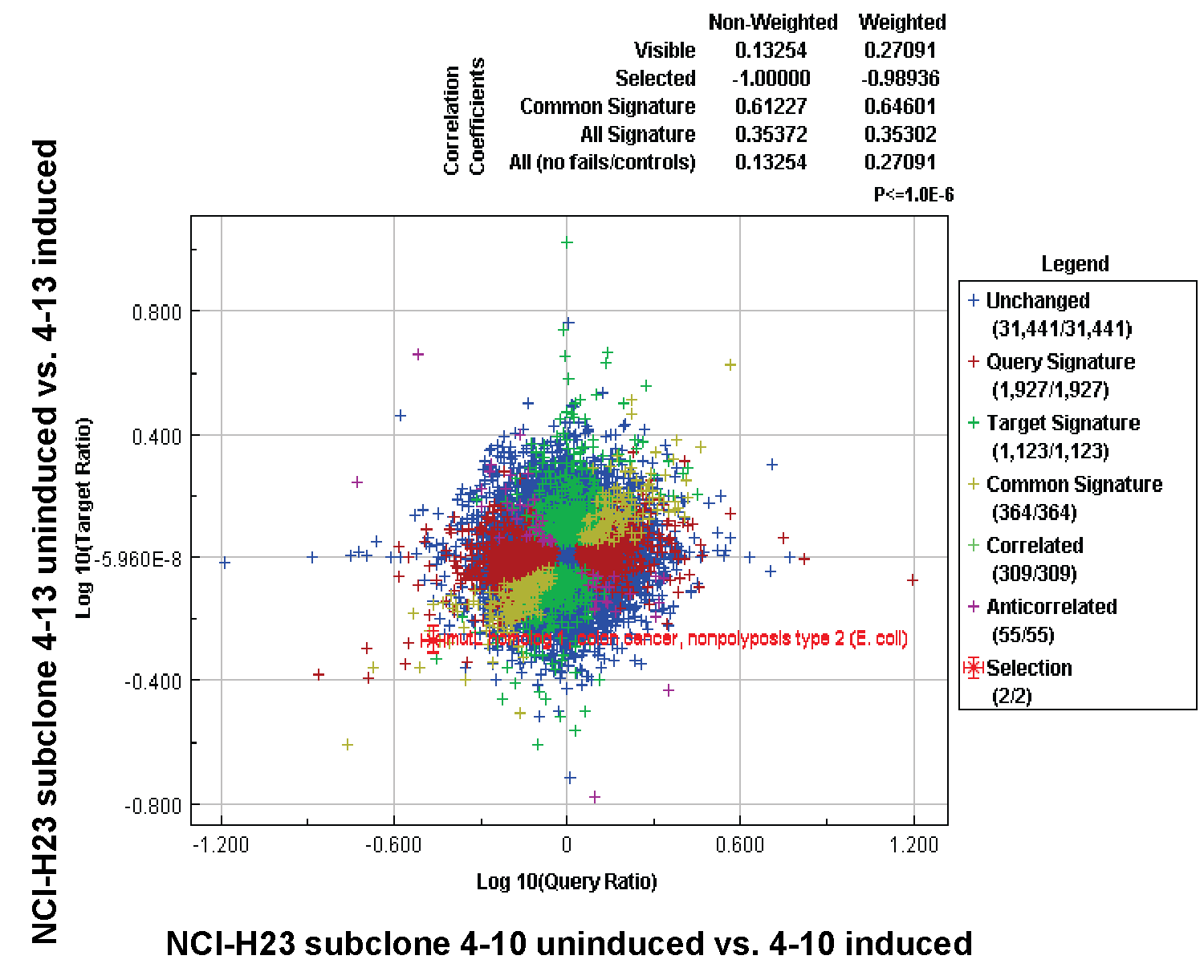

Supplement: Figure S3 — Microarray analysis of NCI-H23 subclones. Total RNA from NCI-H23 subclones grown under uninducing or inducing conditions was labeled and hybridized to whole genome arrays. The gene expression data was exported to Rosetta Resolver and trends compared between the uninduced and induced samples and between the different subclones. The graph shows a comparison of the changes in gene expression for the 4-10 subclone versus (vs.) the 4-13 subclone. Genes with no change in expression level between the subclones are marked in blue. The MLH1 gene, which showed a three-fold decrease in expression in the MLH1-deficient subclones, is indicated in red text. (TIF) [file pone.0078726.s003.tif]

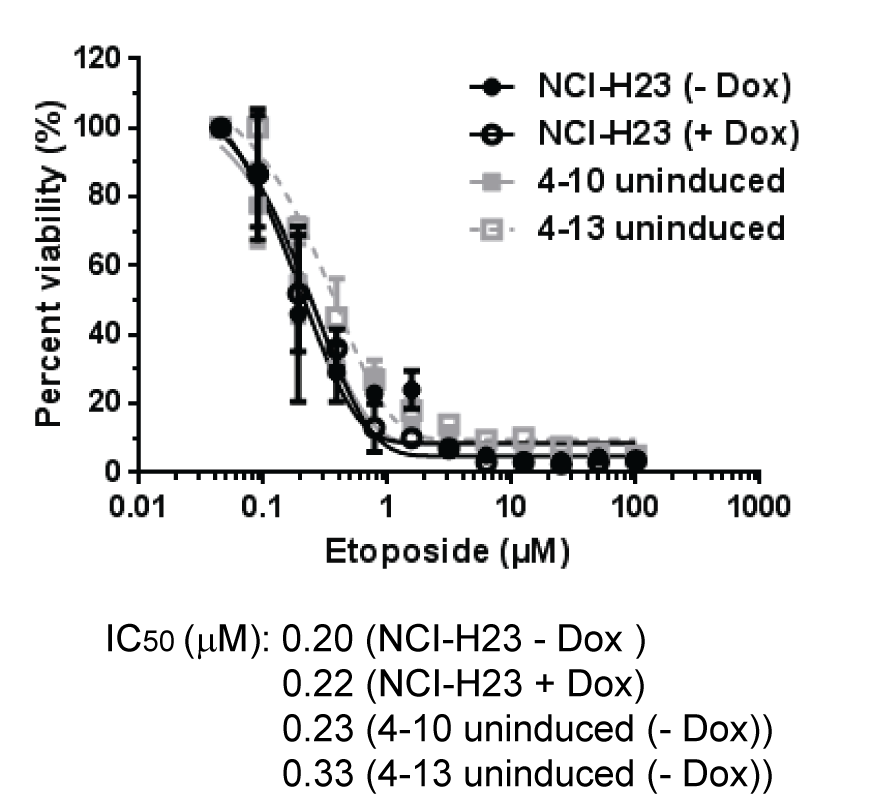

Supplement: Figure S4 — Uninduced NCI-H23 subclones show similar sensitivity to etoposide as the parental cells. NCI-H23 subclones that were uninduced (- Dox) were compared to parental NCI-H23 cells grown in the presence (+ Dox) or absence (- Dox) of doxycycline. Cells were treated at concentrations indicated, and cell viability was assessed after 4d using a Cell Titer-Glo assay. Percent viability of single samples from a representative experiment is shown. Comparison of the IC50 values by t test determined that p=0.34. (TIF) [file pone.0078726.s004.tif]

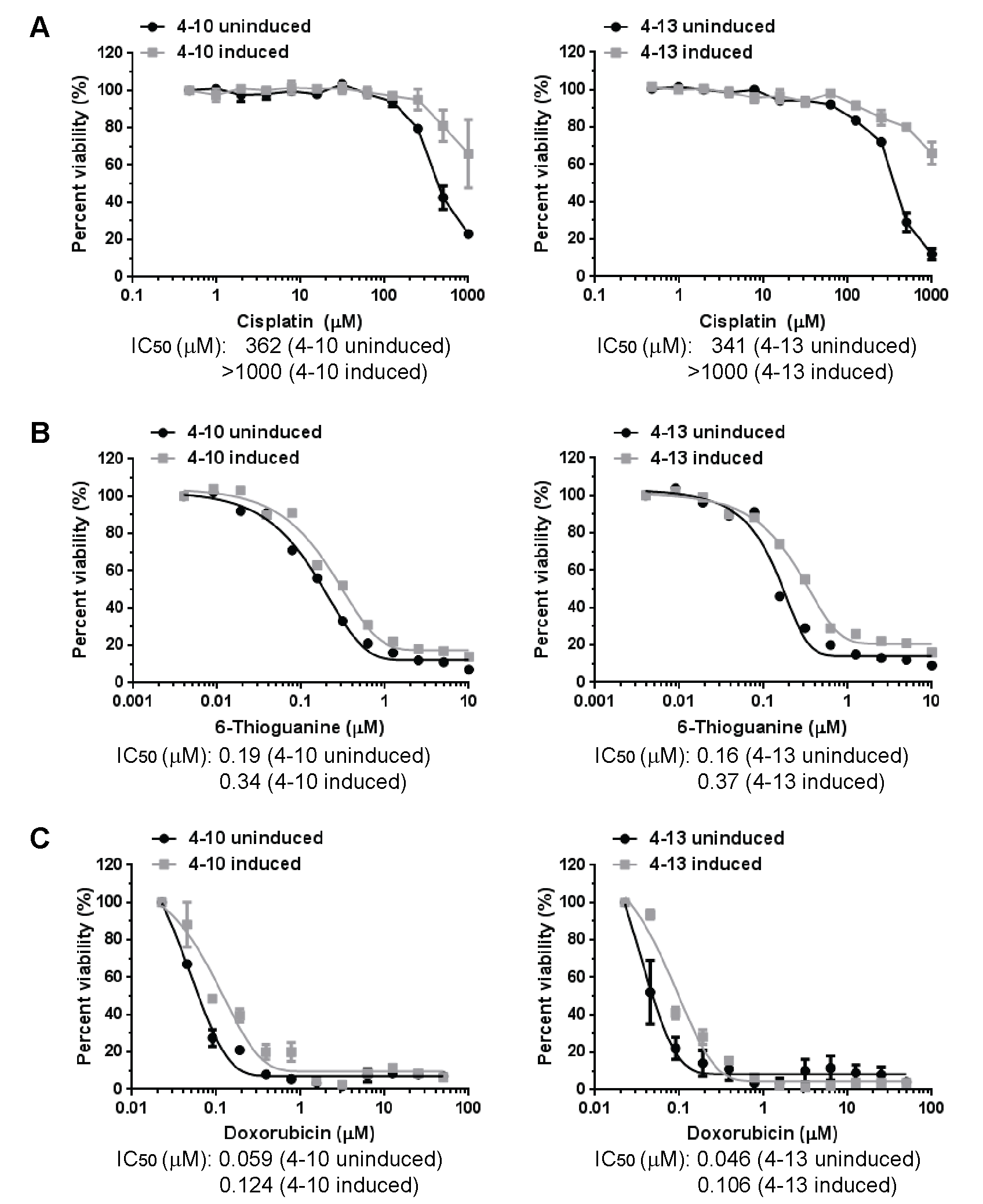

Supplement: Figure S5 — MLH1-deficient NCI-H23 subclones display increased resistance to DNA-damaging drugs. NCI-H23 subclones that were uninduced or induced for MLH1 shRNA were treated with (A) cisplatin, (B) 6-thioguanine or (C) doxorubicin as indicated, and then cell viability was assessed after 4d using a Cell Titer-Glo assay. The graphs indicate the relative survival for duplicate samples from a single experiment. T tests determined the p values as p=0.02, p=0.05 and p=0.04, respectively, for cells treated with cisplatin, 6-thioguanine or doxorubicin. (TIF) [file pone.0078726.s005.tif]

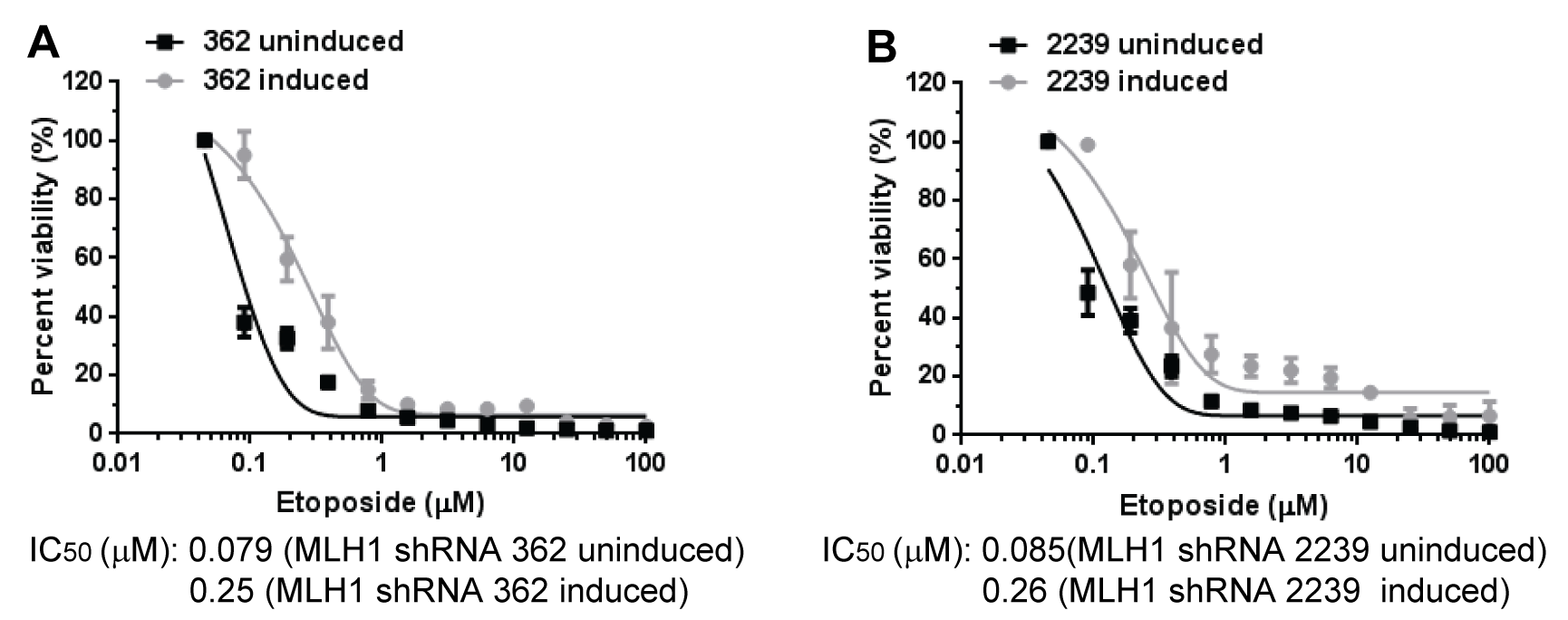

Supplement: Figure S6 — Independent MLH1 shRNA constructs confer differential sensitivity to etoposide. NCI-H23 cells transduced with (A) MLH1 shRNA 362 or (B) MLH1 shRNA 2239 were divided into two cultures, and grown in conditions that were uninduced (MLH1-proficient) or induced for MLH1 shRNA (MLH1-deficient). The cells were treated with etoposide and cell viability was assessed after 4d using a Cell Titer-Glo assay. Percent viability of duplicate samples from a representative experiment is shown. The p value was determined as p=0.01 by t test. (TIF) [file pone.0078726.s006.tif]

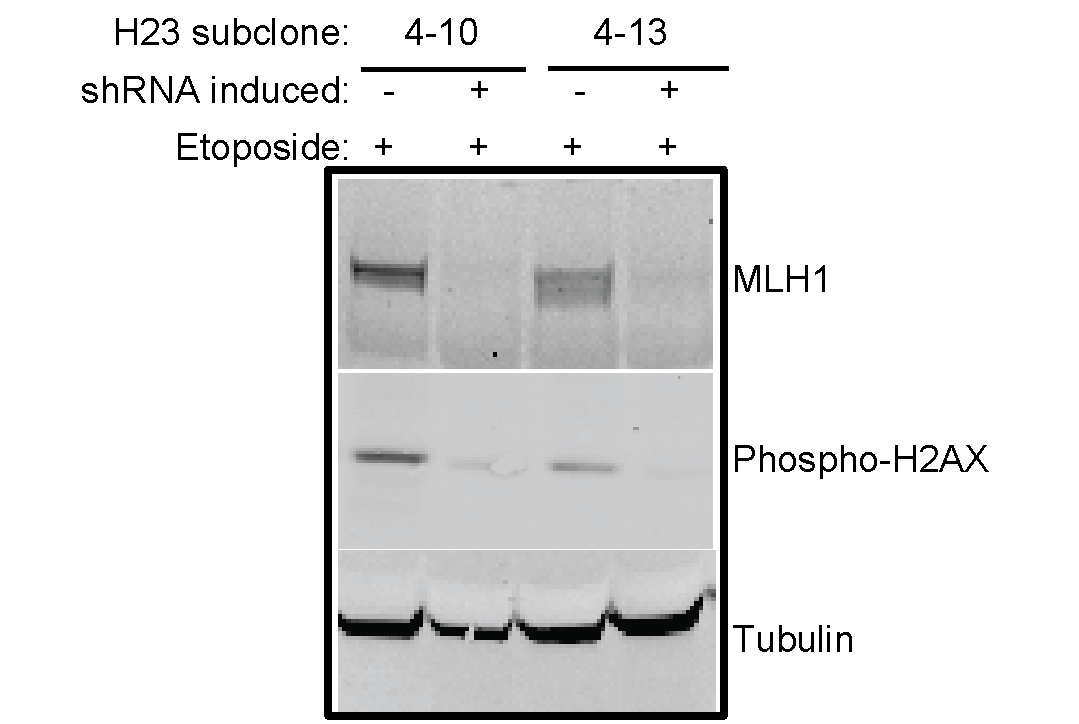

Supplement: Figure S7 — Etoposide treatment induces apoptosis in MMR-proficient NCI-H23 subclones. MLH1-proficient and MLH1-deficient NCI-H23 subclones were treated with 10 µM etoposide for 24h, and then levels of phosphorylated histone H2AX (Phospho-H2AX), a marker for apoptosis, were assessed. MLH1 protein is shown to confirm the cells are MMR-proficient or MMR-deficient. Tubulin levels are shown as a control for protein loading. (TIF) [file pone.0078726.s007.tif]

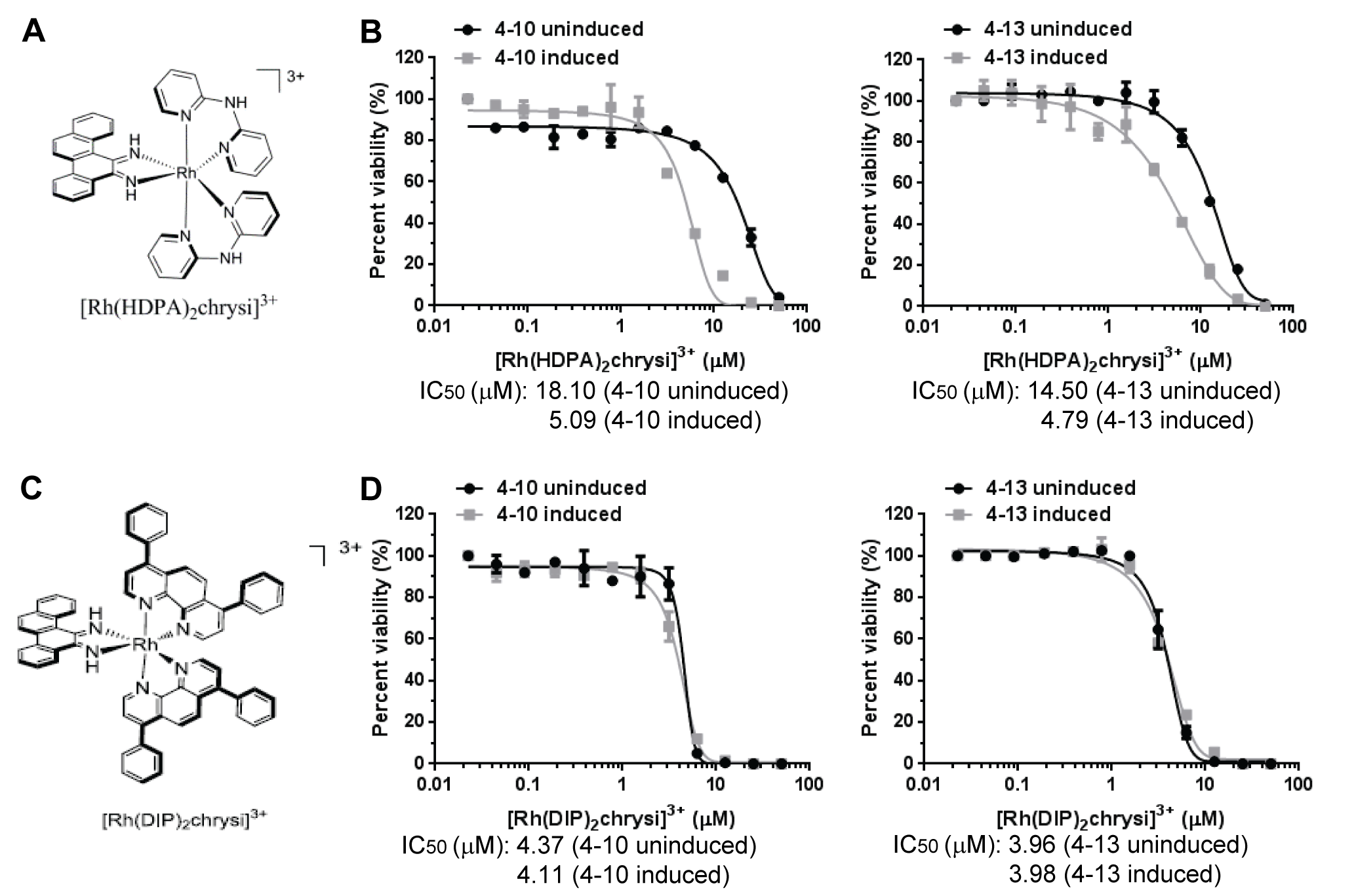

Supplement: Figure S8 — Sensitivity of NCI-H23 subclones to additional rhodium metalloinsertor compounds. (A) Chemical structure of [Rh(HDPA)2chrysi]3+. (B) NCI-H23 subclones that were uninduced or induced for MLH1 shRNA were treated with [Rh(HDPA)2chrysi]3+ as indicated, and cell viability was assessed after 4d using a Cell Titer-Glo assay. A t test determined the p value to be p=0.02. (C) Chemical structure of [Rh(DIP)2chrysi]3+. (D) NCI-H23 subclones that were uninduced or induced for MLH1 shRNA were treated with [Rh(DIP)2chrysi]3+ as indicated, and cell viability was assessed after 4d using a Cell Titer-Glo assay. Percent viability from duplicate samples of a single experiment is shown. A t test determined the p value to be p=0.90. (TIF) [file pone.0078726.s008.tif]

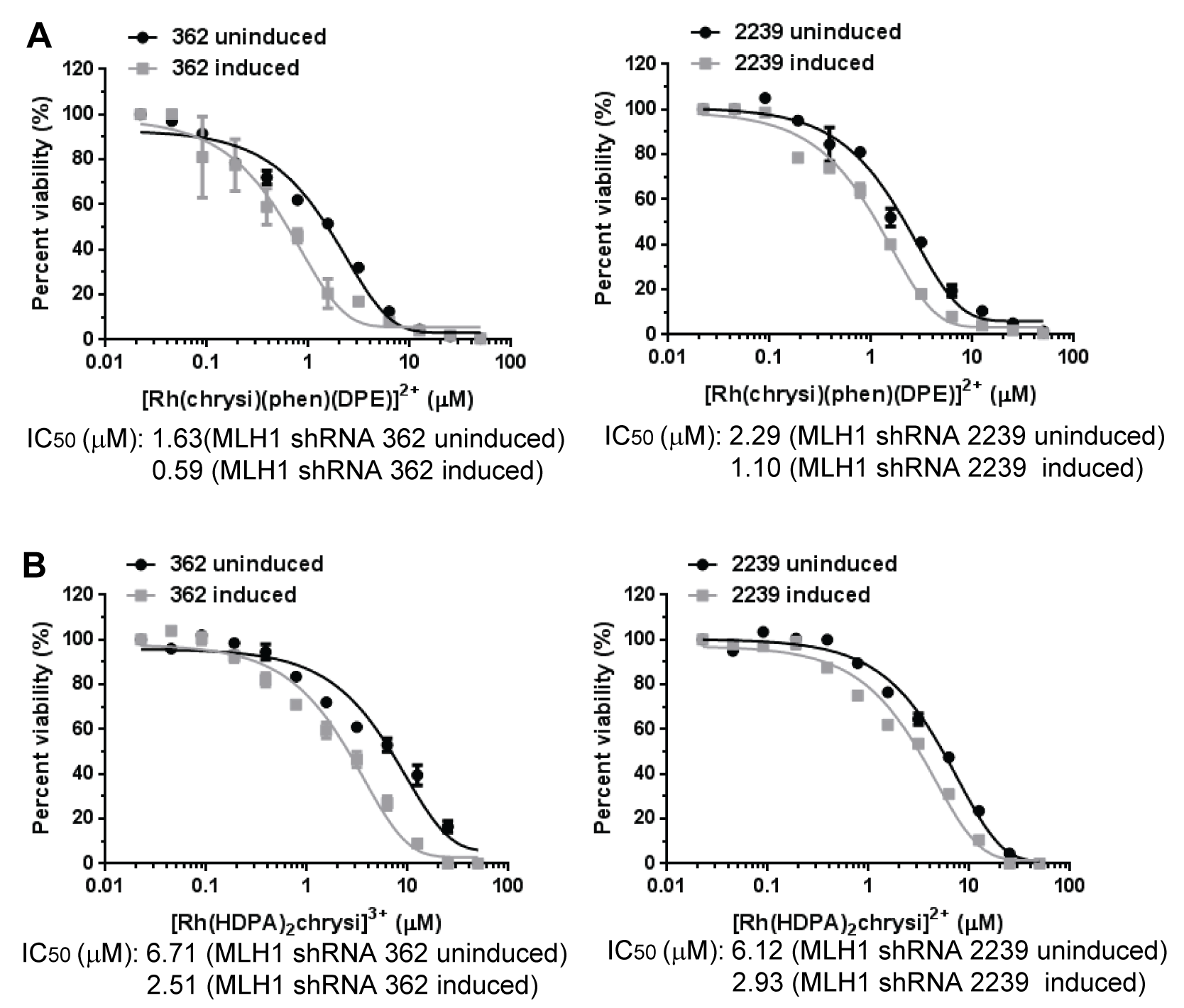

Supplement: Figure S9 — Independent MLH1 shRNA constructs cause preferential sensitivity to rhodium metalloinsertor compounds. NCI-H23 cells transduced with MLH1 shRNA 362 or MLH1 shRNA 2239 were divided into two cultures, and grown in conditions that were uninduced (MLH1-proficient) or induced for MLH1 shRNA (MLH1-deficient). Cells were treated with (A) [Rh(DPE)(phen)chrysi]3+ or (B) [Rh(HDPA)2chrysi]3+ and cell viability was assessed after 4d using a Cell Titer-Glo assay. Percent viability from duplicate samples of a single experiment is shown. The p value was determined as p=0.01 by t test. (TIF) [file pone.0078726.s009.tif]

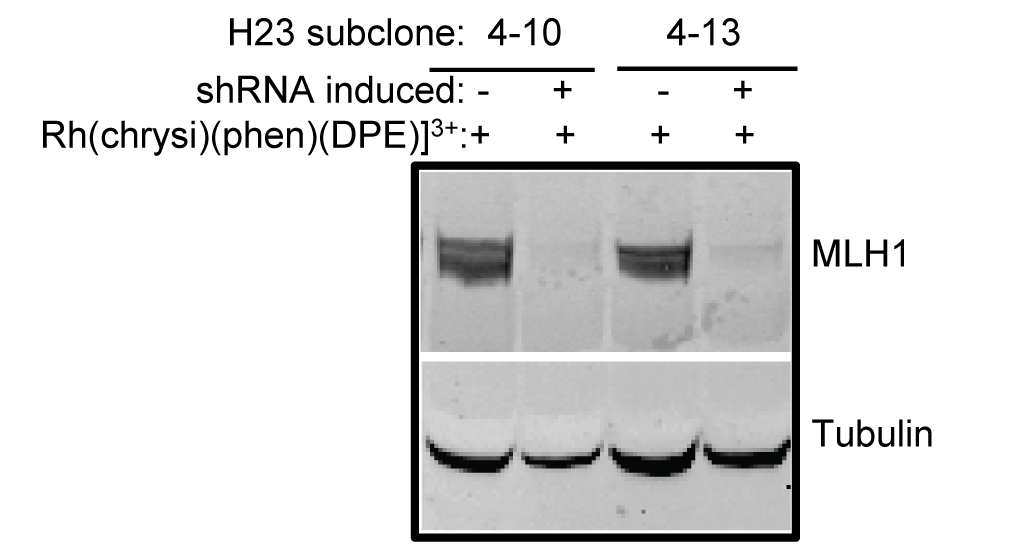

Supplement: Figure S10 — Treatment of NCI-H23 subclones with rhodium metalloinsertor compounds does not alter MSI status. MLH1-proficient and MLH1-deficient NCI-H23 subclones were treated with 5uM [Rh(DPE)(phen)chrysi]3+ for 24h, and then protein lysates were prepared and analyzed for MLH1 protein levels as a marker for MSI. Levels of tubulin were also assessed as a control for protein loading. (TIF) [file pone.0078726.s010.tif]
